# Epidemiologic Survey of Crimean-Congo Hemorrhagic Fever Virus in Suids, Spain

**DOI:** 10.3201/eid3005.240074

**Published:** 2024-05

**Authors:** Mario Frías, Kerstin Fischer, Sabrina Castro-Scholten, Caroline Bost, David Cano-Terriza, Maria Ángeles Risalde, Pelayo Acevedo, Saúl Jiménez-Ruiz, Balal Sadeghi, Martin H. Groschup, Javier Caballero-Gómez, Ignacio García-Bocanegra

**Affiliations:** Universidad de Córdoba, Córdoba, Spain (M. Frías, S. Castro-Scholten, D. Cano-Terriza, M.Á. Risalde, S. Jiménez-Ruiz,^,^ J. Caballero-Gómez, I. García-Bocanegra);; CIBERINFEC, ISCIII, Madrid, Spain (M. Frías, D. Cano-Terriza, M.Á. Risalde, J. Caballero-Gómez, I. García-Bocanegra);; Friedrich-Loeffler-Institut, Greifswald-Insel Riems, Germany (K. Fischer, C. Bost, B. Sadeghi, M.H. Groschup);; Universidad de Castilla La Mancha y Consejo Superior de Investigaciones Científicas (CSIC), Ciudad Real, Spain (P. Acevedo)

**Keywords:** Crimean-Congo hemorrhagic fever virus, arbovirus, CCHFV, Orthonairovirus, Iberian pig, wild boar, viruses, zoonoses, Spain

## Abstract

We conducted a cross-sectional study in wild boar and extensively managed Iberian pig populations in a hotspot area of Crimean-Congo hemorrhagic fever virus (CCHFV) in Spain. We tested for antibodies against CCHFV by using 2 ELISAs in parallel. We assessed the presence of CCHFV RNA by means of reverse transcription quantitative PCR protocol, which detects all genotypes. A total of 113 (21.8%) of 518 suids sampled showed antibodies against CCHFV by ELISA. By species, 106 (39.7%) of 267 wild boars and 7 (2.8%) of 251 Iberian pigs analyzed were seropositive. Of the 231 Iberian pigs and 231 wild boars analyzed, none tested positive for CCHFV RNA. These findings indicate high CCHFV exposure in wild boar populations in endemic areas and confirm the susceptibility of extensively reared pigs to CCHFV, even though they may only play a limited role in the enzootic cycle.

Crimean-Congo hemorrhagic fever virus (CCHFV; genus *Orthonairovirus* family Nairoviridae) is an emerging pathogen mainly transmitted by ticks of the genus *Hyalomma* (*1*,*2*). This arbovirus is the causative agent of Crimean-Congo hemorrhagic fever (CCHF), a severe and lethal zoonotic and hemorrhagic disease in humans (*2*,*3*). In Europe, human cases of CCHF have been traditionally reported only in southeastern countries (*2*,*4*). However, shortly after the virus was detected in Spain (western Europe) in *Hyalomma lusitanicum* ticks collected on red deer (*Cervus elaphus*) in 2010 (*5*), human CCHF clinical cases have been confirmed in western and southwestern Spain since 2013 (*6*–*8*).

Since 2010, endemic circulation of CCHFV has been reported in the Iberian Peninsula in *H. lusitanicum* ticks (*9*,*10*) and red deer, which are the primary host of adult specimens of this tick species (*11*). In this region, red deer populations usually share habitat and natural resources with other susceptible wild ungulate species, such as wild boar (*Sus scrofa*), another important natural host of adult *H. lusitanicum* ticks. This connection, together with the drastic increase of the wild boar population since 1990 in the Iberian Peninsula (*12*), may contribute to the spread and maintenance of the virus in Mediterranean ecosystems. 

Few studies have assessed CCHFV circulation in red deer and wild boar (*11*,*13*–*16*). In addition, those 2 wild ungulate species usually cohabit with extensively managed domestic Iberian pigs; direct and indirect contact between those sympatric species are frequent (*17*). Iberian pig is a breed of the domestic pig autochthonous to the Iberian Peninsula; they are usually reared under extensive management conditions and fattened during the Montanera period (October‒February) by feeding on acorns within large, fenced areas. Most of the Iberian pig farms (80%) are in southwestern Spain, where circulation of CCHFV is high. The European Food Safety Authority (EFSA) has prioritized surveillance of CCHFV in pigs (*18*); however, data on exposure to CCHFV in pigs are lacking (*19*,*20*).The aim of our study was to analyze the circulation of CCHFV in wild boar and sympatric Iberian domestic pigs in southwestern Spain, a hotspot area of CCHFV, where CCHFV-positive ticks are present (*5*,*21*,*22*) and high seroprevalence has been reported in animal hosts (*11*,*14*).

## Methods

### Study Area and Sampling

In this cross-sectional study, we assumed a seroprevalence of 40.6% (*11*) for wild boar and calculated the sample size as 251; we assumed a seroprevalence of 50% (which provides the highest sample size in studies in which seroprevalence is unknown) for a sample of 267 pigs. We calculated those estimates with 95% CI and a desired precision of +6%. Whenever possible, we sampled 14 Iberian pigs in each farm to detect exposure with a 95% probability, assuming a minimum within-farm seroprevalence of 20%.

We sampled wild boar in 35 hunting states between the hunting seasons 2015‒16 and 2020‒21 in 5 provinces of southwestern Spain. In addition, we collected blood samples from Iberian pigs from 18 farms managed under extensive production systems during 2017‒and 2019 in the same study area (Figure). We collected blood samples from pigs at the slaughterhouse and from wild boar by puncture of the cavernous sinus of the dura mater (*23*). We obtained serum samples by blood centrifugation at 400 × *g* for 10 minutes and froze them until serologic and molecular analysis. We recorded epidemiologic information, including sex, age, origin, and year of sampling, from sampled animals whenever possible. We estimated age of wild boar on the basis of tooth replacement patterns. We obtained all Iberian pig samples at the end of the Montanera period.

**Figure:**
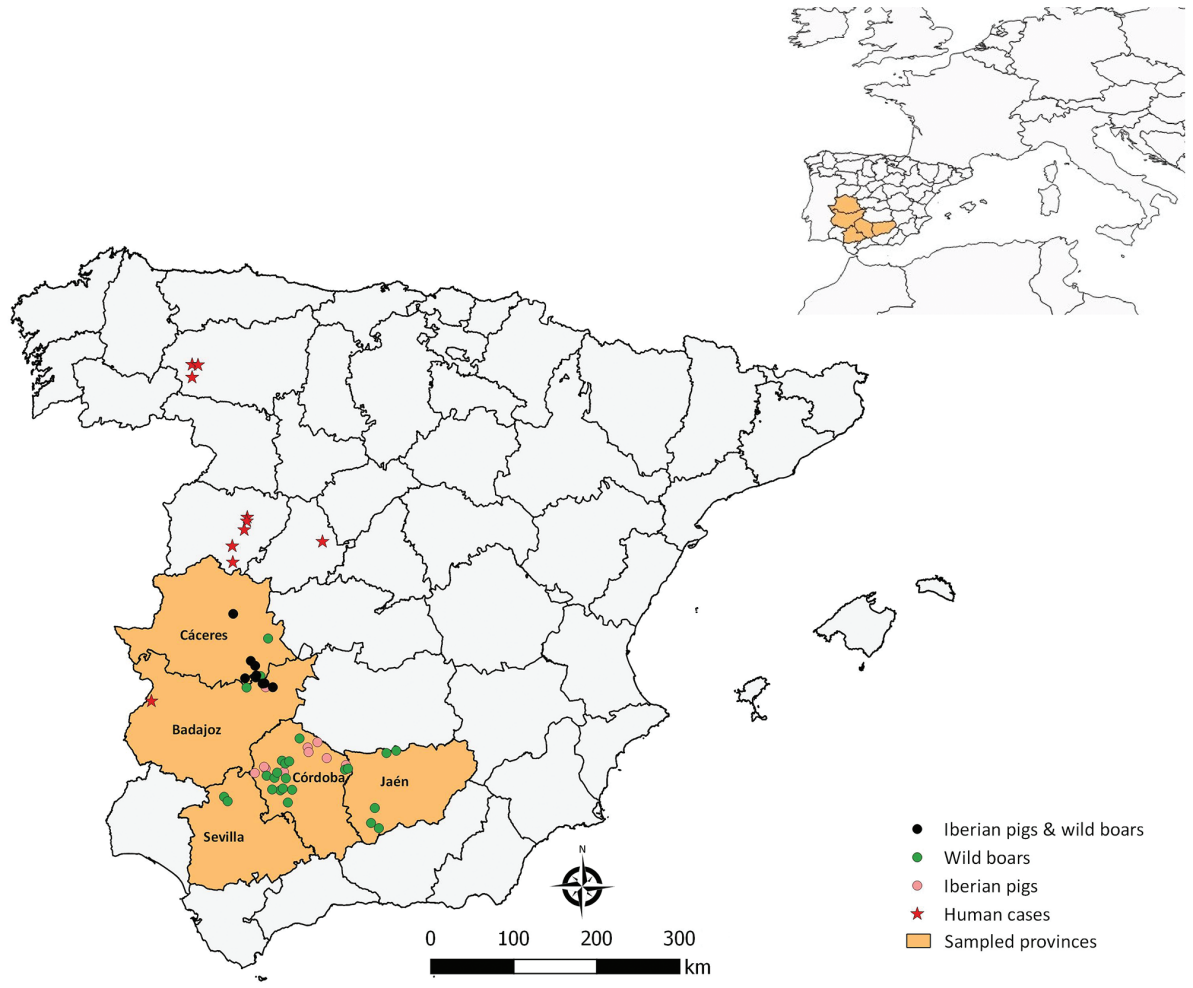
Spatial distribution of samples and human cases notified from epidemiologic survey of Crimean-Congo hemorrhagic fever virus in suids, Spain. Inset map shows location of survey area in western Europe.

### Serologic Methods

We tested for antibodies against CCHFV using 2 different ELISAs, both based on recombinant CCHFV nucleoprotein (N) as described elsewhere (*24*). In brief, the first test was the CCHF Double Antigen Multispecies ELISA (IDvet Screen, https://www.innovative-diagnostics.com), which is based on the CCHFV IbAr10200 strain N of clade III (GenBank accession no. U88410). This commercial test was validated by analyzing several animal species, including pigs (*25*). The second ELISA was an in-house indirect ELISA based on the CCHFV Kosovo Hoti strain N belonging to clade V (GenBank accession no. DQ133507). For this study, we considered samples seropositive if CCHFV antibodies were detected by both ELISAs (double-reactive).

### Molecular Detection

We analyzed a total of 462 serum samples, 231 available from each species, for molecular detection. We extracted RNA from serum samples using the NucleoMag Vet Kit (Macherey-Nagel, https://www.mn-net.com). We assessed the presence of CCHFV RNA by means of QuantiTect Probe RT-PCR Kit (QIAGEN, https://www.qiagen.com) using the multiplex quantitative RT-PCR described elsewhere (*26*), which can amplify a fragment of the short (S) segment of the 6 known CCHFV genotypes.

### Statistical Analysis

We calculated seroprevalence and prevalence of active infection by dividing the number of seropositive animals by ELISA and positive animals by quantitative PCR by the total number of animals tested, using 2-sided exact binomial of 95% CI. We initially assessed associations between serologic results and explanatory variables by species using bivariate analysis with the Pearson χ^2^ or Fisher exact tests, as appropriate. We selected variables with p values <0.10 as potential risk factors. We evaluated collinearity between variables using the Cramer V coefficient, selecting the variable with the highest biologic plausibility if we obtained a correlation coefficient between variables >0.6 and p<0.05. Finally, we evaluated the influence of the selected explanatory variables on CCHFV seropositivity using a generalized linear mixed model (GLMM), assuming a binomial data distribution and the variable municipality as random effect. We considered the Akaike information criterion score for each model to select the most accurate. Variables with p<0.05 were statistically significant. We performed all statistical analyses using RStudio (https://github.com/rstudio/rstudio).

## Results

Of the 518 suids sampled, 71 animals tested positive exclusively through the commercial ELISA, whereas 7 tested positive solely through the in-house assay (*24*). Of note, a total of 113 of the 518 suids sampled showed antibodies against CCHFV by both ELISAs. Thus, the seroprevalence obtained in the preset study was 21.8% (95% CI 18.4%–25.5%; 113/518). By species, 106 (39.7%, 95% CI 34.0%–45.7%) of the 267 wild boar and 7 (2.8%, 95% CI 0.8%–4.8%) of the 251 Iberian pigs analyzed were seropositive (Table 1). We found significantly higher seroprevalence in wild boar than in pigs (relative risk 14.23, 95% CI 6.7–30.0; p<0.001).

**Table 1:** Distribution and seroprevalence of Crimean-Congo hemorrhagic fever in wild boars and extensively raised Iberian pigs, Spain

**Variable**	**Wild boars**				**Iberian pigs**		
	No. positive/no. analyzed	Seroprevalence, % (95% CI)	p value		No. positive/no. analyzed	Seroprevalence, % (95% CI)	p value
Season							
2015–16	10/20	50.0 (28.8–71.1)	0.8885		NA	NA	
2016–17	16/45	35.5 (22.6–50.2)			NA	NA	
2017–18	27/66	40.9 (29.5–53.0)			1/126	0.8 (0–2.3)	0.0123
2018–19	26/63	41.2 (29.6–53.6)			6/77	7.8 (1.8–13.8)	
2019–20	20/52	38.4 (26.0–52.1)			0/48	0 (0–0)	
2020–21	7/21	33.3 (15.9–55.1)			NA	NA	
Province							
Badajoz	7/32	21.9 (7.6–36.2)	0.0119		0/79	0 (0–0.3)	<0.001
Cáceres	20/45	44.4 (30.5–59.1)			6/46	13.0 (3.3–22.8)	
Córdoba	51/100	51.0 (41.2–60.8)			1/126	0.8 (0–2.3)	
Jaén	13/44	29.5 (17.5–44.1)			NA	NA	
Sevilla	15/46	32.6 (19.1–46.2)			NA	NA	
Age							
Yearling	9/45	20.0 (0–26.1)	<0.001		NA	NA	NA
Subadult	23/70	32.9 (21–38.6)			NA	NA	
Adult	72/147	49.0 (41.0–57.0)			NA	NA	
Sex							
M	41/124	33.1 (25.2–41.7)	0.0435		NA	NA	NA
F	64/139	46.0 (37.8–54.3)			NA	NA	

*NA, not applicable.

We found antibodies against CCHFV in wild boar in 26 (70.3%) of the 37 hunting estates sampled and in the 5 provinces analyzed; seroprevalence ranged from 21.9% in Badajoz to 51.0% in Córdoba (Figure). We detected >1 seropositive wild boar in each of the hunting seasons (Table 1). We detected seropositivity in 2 (11.1%) of the 18 pig farms analyzed; within-farm seroprevalence was 5%‒70%. We identified seropositive pigs in 2 provinces sampled; seroprevalence ranged from 0.8% (95% CI 0–2.3%) in Córdoba to 7.8% (95% CI 1.8%–13.8%) in Cáceres (Table 1). We sampled 6/7 seropositive pigs in the province of Cáceres, in the same extensively managed pig farm.

The GLMM model identified age and sex as risk factors potentially associated with human exposure to CCHFV in wild boar (Table 2). We found significantly higher seropositivity in adults (49.0%; odds ratio (OR) 3.9, 95% CI 1.6–9.5) than in yearlings (20.0%). Female (46.0%) wild boar showed 2 times (OR 1.89, 95% CI 1.0–3.4) higher risk of exposure to the virus than males. We identified no risk factor in Iberian pigs by GLMM model. None of the 231 Iberian pigs (0.0%, 95% CI 0.0–1.6%) and 231 wild boars (0.0%, 95% CI 0.0–1.6%) analyzed tested positive to CCHFV RNA presence in their serum.

**Table 2:** Risk factors associated with Crimean-Congo hemorrhagic fever virus seropositivity in wild boar of the Iberian Peninsula, Spain*

Variable	Category	Odds ratio (95% CI)	p value
Age	Yearling	Referent	
	Subadult	2.14 (0.79-5.77)	0.135
	Adult	3.92 (1.61-9.56)	0.003
Sex	M	Referent	
	F	1.89 (1.04-3.42)	0.036

*Analysis by generalized linear mixed model.

## Discussion

Despite extensive data on potential CCHFV hosts and their influence in the maintenance and transmission of this virus (*27*), information on the role of suids in the epidemiology of CCHFV worldwide is very limited. As of February 2024, all surveys conducted worldwide in wild boar have been done in Spain, except for 1 in Turkey, where 2.5% of wild boar were exposed to the virus (*28*). The seroprevalence detected in this species in our study (39.7%) is in accordance with the 40.6% obtained in this species in Doñana National Park in southwestern Spain (*13*) and indicates a high exposure of wild boar populations to CCHFV in this region of Spain. In contrast, lower frequencies of seropositivity were found in northeastern Spain, where 3.2% of wild boar showed antibodies against CCHFV (*15*), and eastern Spain, where 15.3% of wild boar had antibodies (*16*). Those spatial differences found in Spain are consistent with the risk gradient of exposure to CCHFV observed in wild boar on the Iberian Peninsula (*14*) and could be associated with certain climatic factors that can condition the density and abundance of the competent vector species throughout Spain. The presence of *Hyalomma* spp. ticks in northeastern Spain had not been described until recently (*29**,**30*) and in the eastern part of the country was detailed through a study conducted on primary care on bites from these ticks (*31*). In contrast, the presence of *Hyalomma* spp. ticks, including *H. marginatum* and *H. lusitanicum*, has been widely described in southwestern Spain (*9*,*10*,^32^,*33*).

This study provides evidence on the potential role of domestic pigs in the enzootic cycle of CCHFV. As of February 2024, the 2 serosurveys of pigs did not detect antibodies against CCHFV in any of the 25 sampled pigs from India or the 46 sampled in Egypt (*19**, **20*). The seroprevalence obtained (2.8%) confirmed the susceptibility of Iberian pigs to CCHFV infection but indicate a low exposure of extensively raised populations to the virus in a hotspot area, thus suggesting a limited role of this species in the enzootic cycle of CCHFV on the Iberian Peninsula. Nonetheless, it is significantly different from the seroprevalence determined in wild boar. Of note, the period that Iberian pigs are extensively managed (October‒February) does not coincide with the period of main activity of *Hyalomma* spp. ticks (April‒June) (*34*). The pigs are exposed to the vectors for a few months, in contrast to wild boars’ exposure throughout their lives, which could explain the differences in seroprevalence between these species. In addition, this finding could be related to potential differences in susceptibility to viral infection between pigs and wild boar. Future experimental studies can clarify this hypothesis.

In the case of wild boar, we found that age and sex were associated with CCHFV exposure (Table 2). Adults were at 3.6 times higher risk of CCHFV exposure than yearlings, which could be associated with the higher probability of tick bites throughout the life of adult wild boar and with the persistence of CCHFV antibodies over time. This finding is in line with those of Cuadrado-Matías et al. (*13*) and with previous studies that linked the age of cattle with greater seropositivity to CCHFV antibodies (*35**–**37*). On the other hand, female wild boar had significantly higher seroprevalence than males. Previous studies found a higher proportion of female than male wild boar infested by ticks, possibly caused by behavioral differences (*37*). Similarly, other studies have found a higher proportion of CCHFV-seropositive female cattle (*38**,**39*). However, statistically significant differences in CCHFV seropositivity between sexes had not previously been found in the limited serosurveys carried out in wild boar. Additional studies in boar species are needed to evaluate the role of age and sex in CCHFV exposure.

We detected seropositivity every year and in all provinces sampled, indicating an endemic and widespread circulation of CCHFV in southwestern Spain. Of note, yearling animals were seropositive in all of the hunting seasons analyzed except 2020–2021. Although maternal antibodies in yearling mammals cannot be ruled out, our findings denote active circulation during the study period. On the other hand, wild boar from Córdoba province had the highest seroprevalence value; 51% of animals tested seropositive to CCHFV, which was higher than in Badajoz province where human cases have been reported (Figure). In Córdoba, the presence of CCHFV has already been demonstrated by the detection of CCHFV RNA in ticks (*21*,*22*), which would indicate that this region has a high circulation of the virus. Those data highlight the need to carry out studies throughout Spain to establish spatial distribution of CCHFV to promote surveillance and control programs in areas identified as hotspots. On the other hand, 6/7 (85.7%) seropositive pigs were sampled in 2018 in the same extensively managed pig farm in Cáceres. That finding indicates a nonhomogeneous spatiotemporal distribution pattern of the virus in this domestic species, which implies a possible role of the Iberian pig as accidental host rather than true host of CCHFV.

Finally, even though the quantitative PCR used in this study has the capacity to detect all the clades of the virus (*26*), we did not find CCHFV RNA in any of the 462 serum specimens tested. This result is consistent with those previously reported in other mammal species (*40*–*42*) and could be explained by the transient short period of viremia described (<7 days) in small mammals, small ruminants, and hares (*43*,*44*). However, we know of no studies evaluating viremia in suids, which would be necessary to establish their role as natural host of CCHFV.

In conclusion, the results obtained in this study suggest an endemic and widespread circulation of the virus in southwestern Spain. Specifically, these findings indicate high CCHFV exposure in wild boar populations in endemic areas. We report CCHFV exposure in domestic pigs, confirming the susceptibility of this species to the virus, although the low seroprevalence found indicates a limited role in the enzootic cycle of CCHFV in the Iberian and Mediterranean ecosystems. Therefore, we recommend the use of biosecurity measures for high-risk activities with this species to limit exposure to this pathogen. This study highlights the need to develop surveillance programs in suids to evaluate spatiotemporal changes in the circulation of CCHFV to prevent infection in humans.
